# *UGT2B15* single nucleotide polymorphism reduces dabigatran acylglucuronide formation in humans

**DOI:** 10.3389/fphar.2024.1507915

**Published:** 2025-01-09

**Authors:** Jin-Woo Park, Jong-Min Kim, Young Yoon Bang, Kyoung-Ah Kim, Sungwook Yu, Ji-Young Park

**Affiliations:** ^1^ Department of Clinical Pharmacology and Toxicology, Anam Hospital, Korea University College of Medicine, Seoul, Republic of Korea; ^2^ Department of Neurology, Anam Hospital, Korea University College of Medicine, Seoul, Republic of Korea; ^3^ Division of Clinical Pharmacology, Department of Medicine, Vanderbilt University Medical Center, Nashville, TN, United States

**Keywords:** dabigatran, *UGT2B15*, genetic polymorphism, pharmacokinetics, dabigatran acylglucuronide

## Abstract

**Background:**

Dabigatran etexilate (DABE), a prodrug of dabigatran (DAB), is a direct thrombin inhibitor used to prevent ischemic stroke and thromboembolism during atrial fibrillation. The effect of genetic polymorphisms on its metabolism, particularly *UGT2B15*, has not been extensively explored in humans. This study aimed to investigate the effects of *UGT2B15*, *ABCB1*, and *CES1* polymorphisms on the pharmacokinetics of DAB and its acylglucuronide metabolites in healthy subjects.

**Methods:**

A total of 124 healthy males were genotyped for *UGT2B15*, *ABCB1*, and *CES1* polymorphisms. After a single 150 mg dose of DABE, plasma concentrations of total and free DAB, as well as dabigatran acylglucuronide (DABG) were measured using LC-MS/MS. Pharmacokinetic parameters were analyzed using non-compartmental methods, and statistical comparisons were conducted between the genotype groups.

**Results:**

*UGT2B15* c.253G>T significantly affected free DAB pharmacokinetics, with a lower T_max_ and oral clearance in TT genotype (n = 28, *p* < 0.05). For DABG, C_max_ was significantly higher in GG genotypes (n = 32, 42.3 ± 16.3 ng/mL) compared to that in GT (n = 64, 32.4 ± 20.5 ng/mL) and TT (29.7 ± 17.1 ng/mL) genotypes. Similarly, the AUC_all_ of DABG was highest in GG genotypes (327 ± 148.3 ng h·mL^-1^), followed by GT (238.7 ± 166.5 ng h·mL^-1^) and TT (223.3 ± 165.4 ng h·mL^-1^) genotypes (*p* < 0.05). The metabolite-to-parent ratios (m/p ratios) for C_max_ and AUC_all_ were significantly higher in GG and GT genotypes than that in TT genotype. *ABCB1* and *CES1* polymorphisms had no significant impact on the pharmacokinetics of DAB or DABG.

**Conclusion:**

*UGT2B15* polymorphisms were associated with difference in DAB glucuronidation and pharmacokinetics in healthy male participants.

## 1 Introduction

Dabigatran etexilate (DABE), is a prodrug of dabigatran (DAB), a competitive direct thrombin inhibitor widely used in the treatment and prevention of ischemic stroke, atrial fibrillation, thrombus formation, and systemic embolism (Schellong, 2015; Feuring and van Ryn, 2016; Antonijevic et al., 2017; Blair and Keating, 2017). Routine drug monitoring is typically not required for direct oral anticoagulants, including DABE, because of their predictable pharmacokinetics (Härtig et al., 2020). The standard recommended dosage of DABE is 110 or 150 mg twice daily (Connolly et al., 2009; López-López et al., 2017), with adjustments based on individual-factors such as renal function, body weight, age, concurrent use of P-glycoprotein inhibitors, and bleeding risk (Gong and Kim, 2013; Ferri et al., 2022).

As an ester prodrug, DABE undergoes two sequential activation steps to form its active drug, DAB. Initially, DABE is metabolized to dabigatran ethyl ester (M2) by carboxylesterase 2 (CES2) in the intestine (Blech et al., 2008; Antonijevic et al., 2017; Laizure et al., 2022). Subsequently, M2 is converted to DAB by CES1 in the liver. DAB is further metabolized to dabigatran acylglucuronide (DABG) by uridine 5-diphospho (UDP)-glucuronosyltransferase (UGT) enzymes in the liver, with glucuronidation of the carboxylate moiety being the predominant metabolic pathway in humans (Ebner et al., 2010). Among the UGTs, UGT2B15 has been suggested to be the major isoform responsible for DAB glucuronidation (Ebner et al., 2010; Moj et al., 2019).

Given the complexity of the enzymes and transporters involved in DAB metabolism, genetic polymorphisms that affect the function and expression of these enzymes and transporters may contribute to inter-individual variability in DAB metabolism. Several studies have evaluated the clinical impact of *ABCB1* and *CES1* single nucleotide polymorphisms (SNPs) on DAB metabolism and pharmacokinetics (Ji et al., 2021); however, the data generally suggest only minor effects on DAB metabolism (Dimatteo et al., 2016; Ji et al., 2021). The effect of *UGT2B15* SNPs on DAB metabolism, particularly the its impact on DABG formation, has not been extensively explored in humans (Ebner et al., 2010). Given that DABG is a pharmacologically active metabolite, genetic variations affecting its concentration may potentially influence the overall anticoagulant efficacy.

This study primarily aimed to investigate the effect of *UGT2B15* on the pharmacokinetics of DAB in humans, while also considering the roles of *ABCB1* and *CES1* SNPs to provide a more comprehensive understanding of genetic variability’s impact on DABG formation.

## 2 Material and methods

### 2.1 Subjects

This study enrolled 124 male subjects with a mean (±S.D.) age of 25.9 ± 3.7 years (range: 19–38 years), mean weight of 73.1 ± 8.6 kg (range: 54.4–91 kg), and mean height of 175 ± 5.2 cm (range: 160–191 cm). All participants were confirmed to be healthy by a physician through a detailed physical examination, 12-lead electrocardiography, serum biochemistry, hematology, and urinalysis. Exclusion criteria included history or evidence of a hepatic, renal, gastrointestinal, or hematologic abnormality, any other acute or chronic disease, or an allergy to any drug. All subjects were non-smokers, not taking any medication, and provided written informed consent. The study protocol was approved by the Institutional Review Board (IRB) of Anam Hospital, Korea University, Korea (IRB No. 2023AN0054).

### 2.2 Genotyping for *ABCB1*, *CES1*, and *UGT2B15*

To determine the *ABCB1*, *CES1*, and *UGT2B15* genotypes, blood samples were collected from each participant and stored at −20°C until DNA extraction. Genomic DNA was isolated from the peripheral leukocytes. All individuals were genotyped for the c.1236C>T (rs1128503), c.2677C>T(A) (rs2032582), c.3435C>T (rs1045642), and c.2482-2236G>A (rs4148738) alleles of *ABCB1* polymorphisms, c.1168–33A>C (rs2244613) and c.257 + 885T>C (rs8192935) alleles of *CES1* polymorphisms, and c.253G>T (rs1902023) alleles of *UGT2B15* polymorphism through pyrosequencing methods using a PyroMark (Biotage, Uppsala, Sweden), as described previously (Kim et al., 2013a; Kim et al., 2013b; Kim et al., 2014; Park et al., 2022).

### 2.3 Study design

Following an overnight fast, subjects were administered a single oral dose of 150 mg DABE (Pradaxa; Boehringher Ingelheim, Germany) with 240 mL of water. Blood samples were collected in EDTA tubes (Vacutainer; Becton Dickinson, Franklin Lakes, NJ, United States) immediately before drug administration (baseline) and at 0.5, 1, 1.5, 2, 2.5, 3, 4, 6, 8, 10, 12, 24, and 48 h post-administration. Plasma was separated by centrifugation (1977 *g*, 4°C, 15 min) and the samples were stored at −70°C awaiting analysis.

### 2.4 Determination of total DAB, free DAB, and DABG concentrations in plasma samples

Plasma concentrations of DAB were determined using a slightly modified version of a previously validated LC-MS/MS method. The concentration difference between total and free DAB, determined through a deconjugation process, was used to estimate DAB glucuronide levels. This approach followed the method described in the previously literature (Blech et al., 2008). A total of 100 μL of plasma sample was added to a glass tube containing 10 μL of the internal standard, dabigatran-d4 (350 ng/mL). For total DAB determination, 20 μL of potassium hydroxide was added (this step was omitted for free DAB). The mixture was shaken for 15 s, followed by the addition of 400 μL of acetonitrile. The mixture was then vortexed for 1 min, and the organic phase was transferred to a clean glass tube and evaporated to dryness under nitrogen gas flow. The residue was reconstituted with 300 μL of 30% methanol with 1% formic acid. A 3-μL aliquot of this solution was injected onto the LC-MS/MS system which was equipped with a Unison Phenyl column (3 μm, 100 mm × 2.0 mm; Imtakt Corp., Kyoto, Japan). The mobile phase consisted of 10 mM ammonium formate (0.2% formic acid) and methanol in a 60:40 volume ratio at a constant flow rate of 0.2 mL/min. Quantification was performed using multiple reaction monitoring mode, with transitions of *m/z* 472.2→289.1 for DAB and 476.2→293.1 for the internal standard. A linear calibration curve for DAB, ranging from 0.5 to 350 ng/mL, was established, with regression correlation coefficients exceeding 0.9999. Both intra- and inter-day coefficients of variation were maintained below 5%. The concentration difference between total and free DAB was attributed to the DABG concentration.

### 2.5 Pharmacokinetic analysis

The pharmacokinetic parameters for total and free DAB, as well as DABG, were determined using non-compartmental analysis with WinNonlin software (version 8.5.1; Pharsight Corp., Mountain View, CA, United States). Maximum plasma concentration (C_max_) and the time to reach C_max_ (T_max_) were estimated directly from the raw data. The total area under the plasma concentration-time curve (AUC_all_) was calculated using the linear trapezoidal rule. Oral clearance (CL/F) of DAB was estimated using the dose/AUC method. The metabolite-to-parent ratio (m/p ratio) was calculated by dividing the C_max_ and AUC_all_ of DABG by those of free DAB. The C_max_ ratio was calculated by dividing the C_max_ of the metabolite by that of the parent, and the AUC ratio was calculated by dividing the AUC_all_ of the metabolite by that of the parent.

### 2.6 Statistical analysis

The data were expressed as the mean ± standard deviation (SD) in the text and tables. Statistical comparisons between genotype groups (*ABCB1*, *CES1*, and *UGT2B15*) were performed using one-way analysis of variance (ANOVA) or, where appropriate, Kruskal–Wallis one-way ANOVA by rank test. Pharmacokinetic parameters were compared according to each genotype. These tests were chosen based on the normality of the data distribution, which was assessed prior to ANOVA. Multiple *post hoc* comparisons were performed using the normality test to identify significant differences between specific groups. Geometric mean ratios (GMRs) for C_max_ and AUC_all_ were calculated to compare pharmacokinetic parameters among genotypes. GMRs and their 90% confidence intervals were derived using logarithmic transformations, and statistical significance was assessed via t-tests. Heterozygote mutant and homozygote mutant genotypes were analyzed individually, and they were also grouped together to compare against the wild-type genotype, as genetic variations in these genotypes can affect the function of transporters or enzymes involved in drug metabolism. Demographic variables including age, body weight, and height, were used as covariates. However, the interactions between genotype and these covariate were not statistically significant. Data analysis was performed using SAS 9.2 for Windows. Statistical significance was set at *p* < 0.05.

## 3 Results

### 3.1 Genotype frequencies and demographic characteristics

This study analyzed the genotype distributions and allelic frequencies of *ABCB1*, *CES1*, and *UGT2B15* polymorphisms in 124 Korean subjects, along with demographic data such as age, height, and weight (Table 1). The mean age of participants was 25.0 ± 3.7 years, with a mean body weight of 73.1 ± 8.6 kg and height of 175.0 ± 5.2 cm. No significant demographic differences were observed between the genotype groups, except for *ABCB1* c.2677G>T(A) polymorphism, which exhibited five distinct genotype groups and significantly deviation from Hardy-Weinberg equilibrium (*p* = 0.0258), probably due to greater genetic variability among the observed genotypes.

**TABLE 1:** Genotype frequencies of *ABCB1*, *CES1*, and *UGT2B15* genetic polymorphisms in 124 Korean subjects and associated demographic data (Chi-square and P-values calculated based on Hardy-Weinberg Equilibrium [HWE]).

Gene	Genotype	*n*	Age (year)	Height (cm)	Weight (kg)	Allele	Allelic frequency	*χ* ^2^	P-value (HWE)
*ABCB1*									
c.1236C>T (rs1128503)	CC	24	25.3 ± 2.8	173.4 ± 5.8	71.8 ± 10.2	C	0.4274	0.0632	0.7560
	CT	58	25.8 ± 4.0	175.9 ± 5.1	74.3 ± 8.4	T	0.5726		
	TT	42	26.4 ± 3.8	174.5 ± 4.9	72.2 ± 7.9				
	**P-value**		0.5115	0.1107	0.3406				
c.2677G>T(A) (rs2032582)	GG	18	25.0 ± 1.7	174.3 ± 1.2	72.9 ± 4.2	G	0.3911	9.283	0.0258*
	GA	25	25.3 ± 2.9	174.8 ± 5.7	74.6 ± 9.3	T	0.4234		
	GT	36	26.1 ± 3.9	174.6 ± 5.5	72.8 ± 8.3	A	0.1855		
	TA	11	27.0 ± 4.7	175.6 ± 4.9	74.1 ± 8.7				
	AA	5	25.2 ± 2.5	177.4 ± 3.4	73.2 ± 4.7				
	TT	29	26.2 ± 4.1	174.6 ± 5.7	72.7 ± 9.5				
	**P-value**		0.7710	0.8864	0.9185				
c.3435C>T (rs1045642)	CC	52	25.1 ± 2.8	175.1 ± 5.0	72.9 ± 8.4	C	0.621	1.9773	0.1597
	CT	50	26.7 ± 4.4	175.3 ± 5.8	73.7 ± 9.1	T	0.379		
	TT	22	25.9 ± 3.5	173.8 ± 4.6	72.1 ± 8.4				
	**P-value**		0.0959	0.5203	0.7461				
c.2482-2236G>A (rs4148738)	GG	25	25.9 ± 3.4	174.0 ± 4.4	71.8 ± 8.2	G	0.4032	2.6217	0.1054
	GA	50	26.6 ± 4.4	175.4 ± 5.5	73.7 ± 8.9	A	0.5968		
	AA	49	25.2 ± 2.9	175.0 ± 5.4	73.2 ± 8.7				
	**P-value**		0.1723	0.5191	0.6828				
*CES1*									
c.1168–33A>C (rs2244613)	AA	16	25.5 ± 3.1	175.8 ± 6.0	74.8 ± 9.7	A	0.3911	0.8674	0.3517
	AC	65	26.3 ± 3.9	175.3 ± 4.9	74.1 ± 8.6	C	0.6089		
	CC	43	25.4 ± 3.7	174.1 ± 5.5	71.0 ± 8.0				
	**P-value**		0.4152	0.4111	0.1258				
c.257 + 885T>C (rs8192935)	TT	78	25.9 ± 3.8	174.5 ± 5.4	71.8 ± 8.2	T	0.7984	0.0909	0.7631
	TC	42	25.9 ± 3.8	175.7 ± 5.1	75.5 ± 9.4	C	0.2016		
	GG	4	25.0 ± 1.6	175.5 ± 3.5	73.7 ± 3.8				
	**P-value**		0.8870	0.5028	0.0828				
UGT2B15									
c.253G>T (rs1902023)	GG	32	25.6 ± 2.8	175.6 ± 4.0	71.4 ± 7.3	G	0.5161	0.0366	0.8482
	GT	64	26.0 ± 3.9	174.5 ± 5.9	73.2 ± 9.0	T	0.4839		
	TT	28	25.9 ± 4.1	175.2 ± 4.9	74.4 ± 8.9				
	**P-value**		0.9016	0.6071	0.4090				

Bold values and asterisks (*) indicate statistically significant differences (P < 0.05).

### 3.2 Effects of polymorphic *ABCB1*, *CES1* and *UGT2B15* genotypes on free DAB pharmacokinetics

The pharmacokinetics of free DAB, including T_max_, C_max_, AUC_all_, half-life, and CL/F, were evaluated in relation to the genotypes of *ABCB1*, *CES1*, and *UGT2B15* polymorphisms (Table 2). The *UGT2B15* c.253G>T polymorphism was associated with significant differences in the T_max_ (*p* = 0.0479) and CL/F (*p* = 0.0171). Subjects with the GG genotype exhibited slightly longer T_max_ (2.5 ± 0.8 h) compared with the GT (2.2 ± 0.7 h) and TT (2.4 ± 0.6 h) genotypes, but with borderline significance (*p* = 0.047). Wild type versus heterozygote/homozygote mutant anaylsis was also not statistically significant. Although CL/F appeared lower in the TT genotype group (177.2 ± 81.5 L/h) compared to the GT (247.9 ± 155.5 L/h) and GG (185.8 ± 90.9 L/h) groups, there was no consistent allelic dose-effect relationship observed to suggest a clear genotype-related trend. No significant differences in DAB pharmacokinetics were observed in the *ABCB1* and *CES1* polymorphisms. Additionally, no significant differences in GMR values for DAB were observed among the UGT2B15 genotypes (Table 3).

**TABLE 2:** Comparisons of pharmacokinetic variables of free DAB, DABG, and metabolite-to-parent ratios (m/p ratios) by *UGT2B15* genetic polymorphisms.

Parameters	Substance	Wild type (W)	Heterozygous (H)	Homozygous mutants (M)	H and M	P-value	
		GG (n = 32)	GT (n = 64)	TT (n = 28)	GT, TT (n = 92)	W vs. H vs. M	W vs. H and M
T_max_ (h)	DAB	2.5 ± 0.8	2.2 ± 0.7	2.4 ± 0.6	2.2 ± 0.7	0.0479*	0.0654
	DABG	2.2 ± 0.7	2.2 ± 0.7	2.2 ± 0.8	2.2 ± 0.7	0.9989	0.9869
C_max_ (ng/mL)	DAB	108.1 ± 37.6	95.4 ± 51.9	111.4 ± 38.2	100.3 ± 48.5	0.2184	0.4102
	DABG	42.3 ± 16.3	32.4 ± 20.5	29.7 ± 17.1	31.6 ± 19.5	0.0188*^a,b^	0.0059*
AUC_all_ (ng·h·mL^-1^)	DAB	927.8 ± 325	803.2 ± 430.2	972.5 ± 360.1	854.7 ± 415.6	0.1098	0.3681
	DABG	327 ± 148.3	238.7 ± 166.5	223.3 ± 165.4	234 ± 165.4	0.0207*^a,b^	0.0058*
Half-life (h)	DAB	8.6 ± 0.9	9.1 ± 1.5	8.9 ± 1.5	9.1 ± 1.5	0.2698	0.1318
	DABG	10.2 ± 1.6	10.6 ± 2.5	10.4 ± 3.1	10.6 ± 2.7	0.6692	0.4107
CL/F (L/h)	DAB	185.8 ± 90.9	247.9 ± 155.5	177.2 ± 81.5	226.4 ± 140.6	0.0171*^c^	0.1309
	DABG	-	-	-	-	-	-
m/p ratio, C_max_	DABG/DAB	0.4 ± 0.1	0.4 ± 0.2	0.3 ± 0.1	0.3 ± 0.1	0.0217*^b,c^	0.004*
m/p ratio, AUC_all_		0.4 ± 0.1	0.3 ± 0.2	0.2 ± 0.1	0.3 ± 0.2	0.0003*^b,c^	0.0088*

C_max_, maximum concentration; T_max_, time required to reach the maximum concentration; AUC_all_, total area under the plasma concentration–time curve. *P < 0.05; ^a^P < 0.05 between W and H; ^b^P < 0.05 between W and M; ^c^P < 0.05 between H and M.

Bold values and asterisks (*) indicate statistically significant differences (P < 0.05).

**TABLE 3:** Geometric Mean Ratio (GMR) Comparisons of C_max_ and AUC_all_ for DAB, DABG, and metabolite-to-parent Ratios (m/p ratios) by *UGT2B15* genetic polymorphisms (GT and TT were compared to GG, and TT was compared to GT).

Substance	Parameter	Comparison	GMR	90% CI (Lower)	90% CI (Upper)	P-Value
DAB	C_max_	GG vs. GT	0.83	0.68	1.01	0.1861
		GG vs. GT	1.04	0.86	1.26	0.7124
		GT vs. TT	1.26	0.99	1.59	0.0941
	AUC_all_	GG vs. GT	0.80	0.65	0.98	0.1085
		GG vs. GT	1.05	0.87	1.26	0.6823
		GT vs. TT	1.32	1.04	1.66	0.0504
DABG	C_max_	GG vs. GT	0.98	0.78	1.23	0.8727
		GG vs. GT	0.70	0.56	0.87	0.0059*
		GT vs. TT	0.72	0.56	0.92	0.0264*
	AUC_all_	GG vs. GT	0.98	0.78	1.22	0.8698
		GG vs. GT	0.65	0.52	0.82	0.0040*
		GT vs. TT	0.67	0.52	0.86	0.0111*
m/p ratio	C_max_	GG vs. GT	1.18	1.01	1.37	0.0898
		GG vs. GT	0.67	0.57	0.78	<0.0001*
		GT vs. TT	0.57	0.52	0.63	<0.0001*
	AUC_all_	GG vs. GT	1.23	1.06	1.42	0.0379*
		GG vs. GT	0.62	0.54	0.72	<0.0001*
		GT vs. TT	0.51	0.46	0.56	<0.0001*

C_max_, maximum concentration; AUC_all_, total area under the plasma concentration–time curve; GG, wild type; GT, heterozygote mutant; TT, homozygote mutant.

Bold values and asterisks (*) indicate statistically significant differences (P < 0.05).

### 3.3 Effects of polymorphic *ABCB1*, *CES1,* and *UGT2B15* genotypes on DABG pharmacokinetics

**FIGURE 1:**
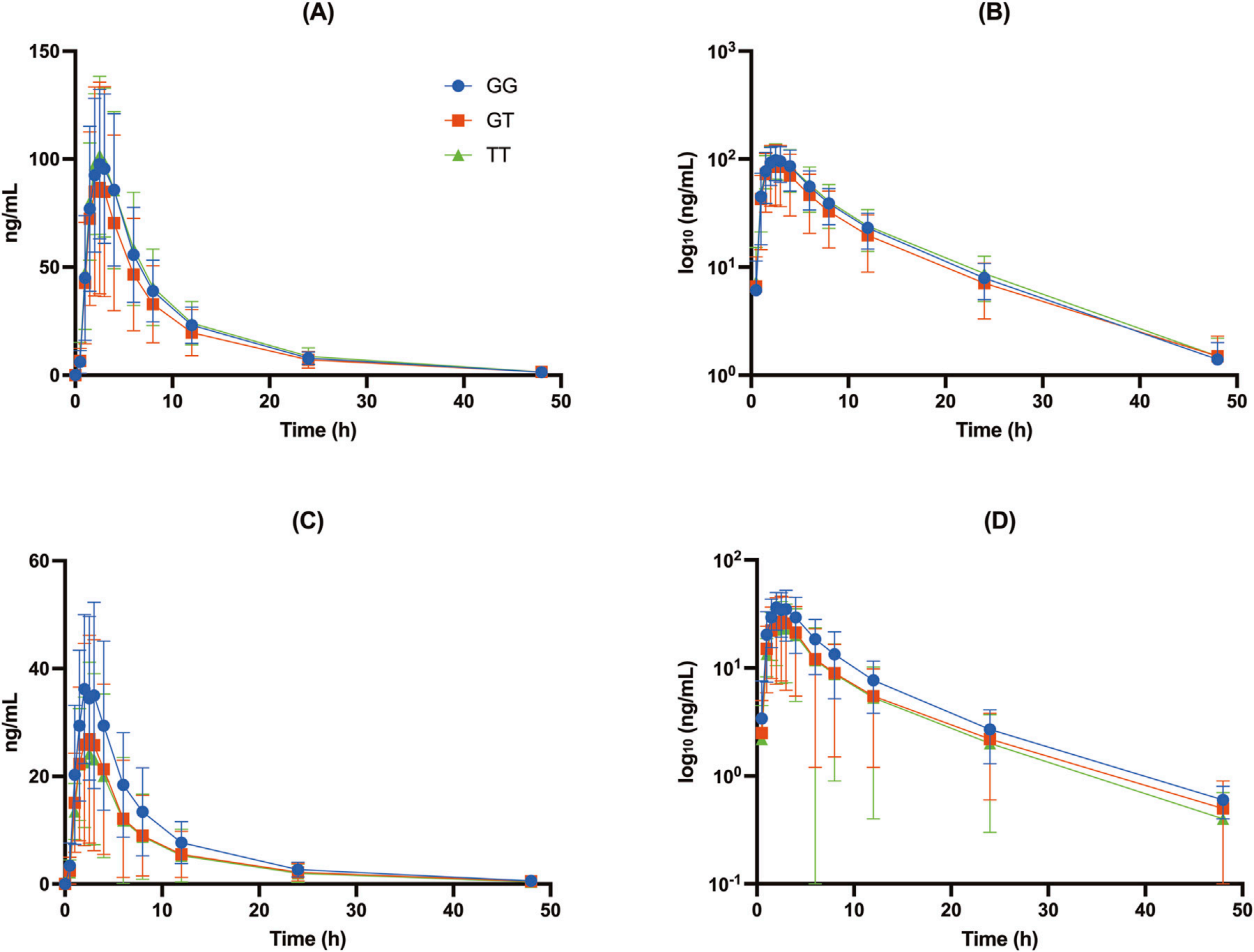
Concentration-time profiles of DAB and DABG for the *UGT2B15* genotype (rs1902023): CC (wild-type), CA (heterozygote mutant), and AA (homozygote mutant). **(A)** Free DAB plasma concentrations (ng/mL); **(B)** log_10_-transformed free DAB plasma concentrations (log_10_ [ng/mL]); **(C)** DABG plasma concentrations (ng/mL); **(D)** log_10_-transformed DABG plasma concentrations (log_10_ [ng/mL]).

**FIGURE 2:**
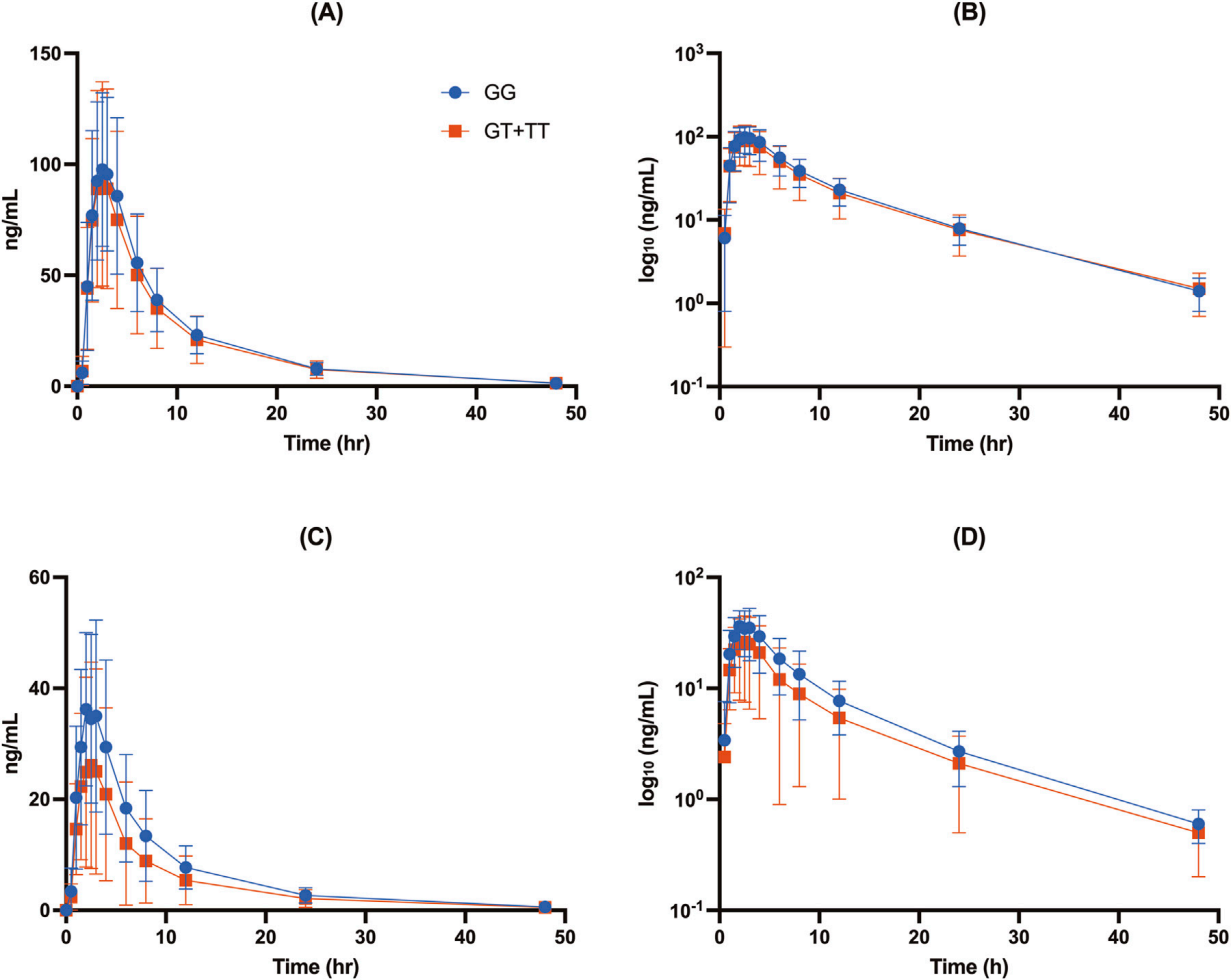
Concentration-time profiles of free DAB **(A, B)** and DABG **(C, D)** stratified by *UGT2B15* genotype (rs1902023) groups: GG (wild-type), GT (heterozygote mutant), and TT (homozygote mutant). Data are presented as mean ± SD for plasma concentrations **(A, C)** and log_10_-transformed plasma concentrations **(B, D)** for each genotype group.

**FIGURE 3:**
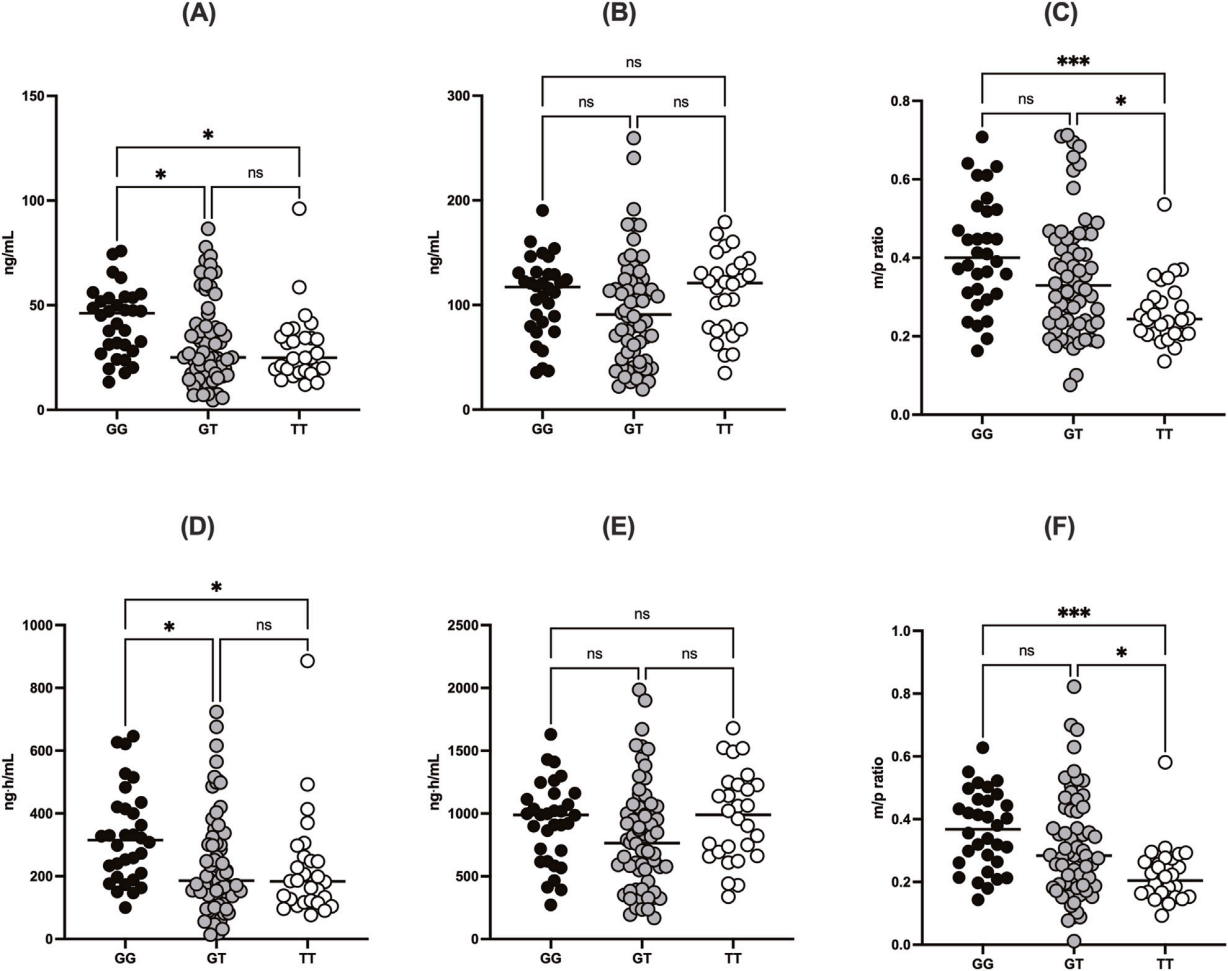
Comparisons of **(A)** C_max_ of DABG, **(B)** C_max_ of free DAB, **(C)** m/p ratio for C_max_, **(D)** AUC_all_ of DABG, **(E)** AUC_all_ of free DAB, and **(F)** m/p ratio for AUC_all_ according to *UGT2B15* genotype (rs1902023). Data are stratified by genotype groups: GG (wild-type), GT (heterozygote mutant), and TT (homozygote mutant). * indicates *p* < 0.05.

The *UGT2B15* c.253G>T polymorphism significantly affected the pharmacokinetics of DABG (Table 2, 3; Figures 1–3). Subjects with the GG genotype exhibited higher C_max_ and AUC_all_ values than those with GT or TT genotypes (*p* = 0.0188 and *p* = 0.0207, respectively). GMR analyses revealed significantly lower C_max_ and AUC_all_ values in the TT genotype compared to the GG genotype (C_max_ GMR: 0.70, *p* = 0.0059; AUC_all_ GMR: 0.65, *p* = 0.004). Similarly, GT vs. TT comparisons showed reductions in both C_max_ (GMR: 0.72, *p* = 0.0264) and AUC_all_ (GMR: 0.67, *p* = 0.0111). These findings indicate that the *UGT2B15* c.253G>T polymorphism plays a critical role in DABG metabolism, potentially affecting drug exposure. No significant differences were observed for the *ABCB1* and *CES1* polymorphisms.

### 3.4 Effects of polymorphic *ABCB1*, *CES1,* and *UGT2B15* genotypes on m/p ratio

The *UGT2B15* c.253G>T polymorphism was also associated with significant differences in the m/p ratio of DABG (Table 2, 3; Figure 3). Specifically, significant differences were observed in the C_max_ (*p* = 0.0217) and AUC_all_ (*p* = 0.0003) values between the GG, GT, and TT genotypes. GMR analyses revealed significantly lower m/p ratio values for both C_max_ and AUC_all_ in the TT genotype compared to the GG genotype (C_max_ GMR: 0.57, *p* < 0.0001, AUC_all_ GMR: 0.51, *p* < 0.0001). Similarly, GT vs. TT comparisons demonstrated reductions in C_max_ (GMR: 0.57, *p* < 0.0001) and AUC_all_ (GMR: 0.51, *p* < 0.0001). These findings suggest a genotype-dependent effect on the conversion of DAB to its acylglucuronide form, highlighting the potential influence of *UGT2B15* polymorphism on DAB metabolism.

## 4 Discussion

This study demonstrated that a *UGT2B15* polymorphism, specifically c.253G>T, significantly influences the pharmacokinetics of DABG and the m/p ratio, supporting the hypothesis that genetic variability in *UGT2B15* plays a key role in DAB metabolism. DAB undergoes glucuronidation primarily by *UGT2B15*, which converts the active form of the drug into its acylglucuronide metabolite (Ebner et al., 2010).

Our data showed that individuals with the GG genotype had significantly higher DABG concentrations than those with the GT or TT genotypes, suggesting that this polymorphism influenced both the rate (C_max_) and extent (AUC_all_) of glucuronidation. Specifically, the C_max_ of DABG was 42.3 ± 16.3 ng/mL in the GG genotypes, compared with 32.4 ± 20.5 ng/mL in the GT and 29.7 ± 17.1 ng/mL in the TT genotypes (*p* < 0.05) (Table 2; Figures 1, 2). The AUC_all_ of DABG followed a similar trend, with values of 327 ± 148.3 ng h·mL^**-1**^ in the GG genotypes, 238.7 ± 166.5 ng h·mL^**-1**^ in the GT genotypes, and 223.3 ± 165.4 ng h·mL^**-1**^ in the TT genotypes, highlighting the significant impact of the *UGT2B15* c.253G>T polymorphism on glucuronidation efficiency. Furthermore, the observed differences in the m/p ratios provide additional evidence to support this hypothesis. The m/p ratios for both C_max_ and AUC_all_ were significantly higher in the GG and GT genotypes than in the TT genotype (Table 2; Figure 3), indicating more efficient conversion of DAB to its acylglucuronide form in the GG and GT genotypes. Conversely, the reduced m/p ratio among individuals with the TT genotype suggests that this polymorphism impairs the conversion of DAB to its acylglucuronide form, further confirming the role of *UGT2B15* in DAB metabolism. A lower m/p ratio in TT genotypes (indicative of less extensive metabolism) aligns with the hypothesis that the *UGT2B15* polymorphism diminishes enzyme function, resulting in reduced glucuronidation capacity. Indeed, the role of *UGT2B15* in sipoglitazar glucuronidation activity was experimentally demonstrated previously (Nishhara, 2013). The UGT2B15 variant exhitibed 2-fold reduction in intrinsic clearance for sipoglitazar when compared to the wild-type. Taken together, our results suggest that UGT2B15 is responsible for DAB glucuronidation, and the UGT2B15 polymorphism in humans likely decreases DABG formation due to the loss of function associated with this mutation.

In contrast, *ABCB1* and *CES1* polymorphisms did not significantly affect DAB metabolism in this study Supplementary Tables S1- S3. No statistically significant differences in the pharmacokinetic parameters were observed between the *ABCB1* and *CES1* genotypes, suggesting that these genetic variations do not play a major role in DAB metabolism.

This study had several limitations. First, it was conducted on healthy adult males, which may limit the generalizability of the findings to broader patient populations, including females, older individuals, and those with comorbid conditions. However, limiting the study to specific demographic variables allowed for the control of potential confounders (Park et al., 2021). Second, this study only examined the effects of a single-dose administration of DAB, leaving the impact of *UGT2B15* polymorphisms on long-term treatment and real-world clinical settings remain to be determined. Third, although we identified the effects of genetic polymorphisms on DAB metabolism, we did not assess the clinical outcomes associated with these genetic variations, such as bleeding risk or therapeutic efficacy. Future studies should investigate these clinical endpoints to provide a more comprehensive understanding of the effects of *UGT2B15* polymorphisms.

In conclusion, this study provides preliminary evidence that the UGT2B15 c.253G>T polymorphism may influence the pharmacokinetics of DABG in humans, particularly in glucuronidation and the m/p ratio, suggesting a potential role for genetic variability in individual responses to DAB therapy. However, further studies are necessary to assess their potential impact on clinical outcomes and to evaluate the generalizability of these findings to the broader population.

## Data Availability

The original contributions presented in the study are publicly available. This data can be found here: https://doi.org/10.6084/m9.figshare.28040075.v1.

## References

[B1] Antonijevic N. M. Zivkovic I. D. Jovanovic L. M. Matic D. M. Kocica M. J. Mrdovic I. B. (2017). Dabigatran - metabolism, pharmacologic properties and drug interactions. Curr. Drug Metab. 18, 622–635. 10.2174/1389200218666170427113504 28460624

[B2] Blair H. A. Keating G. M. (2017). Dabigatran etexilate: a review in nonvalvular atrial fibrillation. Drugs 77, 331–344. 10.1007/s40265-017-0699-z 28185082

[B3] Blech S. Ebner T. Ludwig-Schwellinger E. Stangier J. Roth W. (2008). The metabolism and disposition of the oral direct thrombin inhibitor, dabigatran, in humans. Drug Metab. Dispos. 36, 386–399. 10.1124/dmd.107.019083 18006647

[B4] Connolly S. J. Ezekowitz M. D. Yusuf S. Eikelboom J. Oldgren J. Parekh A. (2009). Dabigatran versus warfarin in patients with atrial fibrillation. N. Engl. J. Med. 361, 1139–1151. 10.1056/NEJMoa0905561 19717844

[B5] Dimatteo C. D’Andrea G. Vecchione G. Paoletti O. Cappucci F. Tiscia G. L. (2016). Pharmacogenetics of dabigatran etexilate interindividual variability. Thromb. Res. 144, 1–5. 10.1016/j.thromres.2016.05.025 27261537

[B6] Ebner T. Wagner K. Wienen W. (2010). Dabigatran acylglucuronide, the major human metabolite of dabigatran: *in vitro* formation, stability, and pharmacological activity. Drug Metab. Dispos. 38, 1567–1575. 10.1124/dmd.110.033696 20551237

[B7] Ferri N. Colombo E. Tenconi M. Baldessin L. Corsini A. (2022). Drug-drug interactions of direct oral anticoagulants (DOACs): from pharmacological to clinical practice. Pharmaceutics 14, 1120. 10.3390/pharmaceutics14061120 35745692 PMC9229376

[B8] Feuring M. van Ryn J. (2016). The discovery of dabigatran etexilate for the treatment of venous thrombosis. Expert Opin. Drug Discov. 11, 717–731. 10.1080/17460441.2016.1188077 27159158

[B9] Gong I. Y. Kim R. B. (2013). Importance of pharmacokinetic profile and variability as determinants of dose and response to dabigatran, Rivaroxaban, and apixaban. Can. J. Cardiol. 29 (Suppl. ment), S24–S33. 10.1016/j.cjca.2013.04.002 23790595

[B10] Härtig F. Poli S. Ebner M. Birschmann I. Kuhn J. Ziemann U. (2020). Monitoring of low dabigatran concentrations: diagnostic performance at clinically relevant decision thresholds. J. Thromb. Thrombolysis. 49, 457–467. 10.1007/s11239-019-01981-z 31691890

[B11] Ji Q. Zhang C. Xu Q. Wang Z. Li X. Lv Q. (2021). The impact of ABCB1 and CES1 polymorphisms on dabigatran pharmacokinetics and pharmacodynamics in patients with atrial fibrillation. Br. J. Clin. Pharmacol. 87, 2247–2255. 10.1111/bcp.14646 33179295

[B12] Kim K. A. Joo H. J. Lee H. M. Park J. Y. (2013a). SLCO2B1 genetic polymorphisms in a Korean population: pyrosequencing analyses and comprehensive comparison with other populations. Mol. Biol. Rep. 40, 4211–4217. 10.1007/s11033-013-2502-x 23666051 PMC3685710

[B13] Kim K. A. Song W. G. Lee H. M. Joo H. J. Park J. Y. (2013b). Effect of P2Y1 and P2Y12 genetic polymorphisms on the ADP-induced platelet aggregation in a Korean population. Thromb. Res. 132, 221–226. 10.1016/j.thromres.2013.06.020 23849096

[B14] Kim K. A. Song W. G. Lee H. M. Joo H. J. Park J. Y. (2014). Multiplex Pyrosequencing method to determine CYP2C9*3, VKORC1*2, and CYP4F2*3 polymorphisms simultaneously: its application to a Korean population and comparisons with other ethnic groups. Mol. Biol. Rep. 41, 7305–7312. 10.1007/s11033-014-3617-4 25069408

[B15] Laizure S. C. Chen F. Farrar J. E. Ali D. Yang B. Parker R. B. (2022). Carboxylesterase-2 plays a critical role in dabigatran etexilate active metabolite formation. Drug Metab. Pharmacokinet. 47, 100479. 10.1016/j.dmpk.2022.100479 36375226

[B16] López-López J. A. Sterne J. A. C. Thom H. H. Z. Higgins J. P. T. Hingorani A. D. Okoli G. N. (2017). Oral anticoagulants for prevention of stroke in atrial fibrillation: systematic review, network meta-analysis, and cost effectiveness analysis. BMJ 359, j5058. 10.1136/bmj.j5058 29183961 PMC5704695

[B17] Moj D. Maas H. Schaeftlein A. Hanke N. Gómez-Mantilla J. D. Lehr T. (2019). A comprehensive whole-body physiologically based pharmacokinetic model of dabigatran etexilate, dabigatran and dabigatran glucuronide in healthy adults and renally impaired patients. Clin. Pharmacokinet. 58, 1577–1593. 10.1007/s40262-019-00776-y 31104266

[B18] Nishhara M. (2013). UDP-glucuronosyltransferase 2B15 (UGT2B15) is the major enzyme responsible for sipoglitazar glucuronidation in humans: retrospective identification of the UGT isoform by *in vitro* analysis and the effect of UGT2B15*2 mutation. Drug Metab. Pharmacokinet. 10.2133/dmpk.dmpk-13-rg-004 23648677

[B19] Park J. W. Chung H. Kim K. A. Kim J. M. Park I. H. Lee S. (2021). ABCG2 single nucleotide polymorphism affects imatinib pharmacokinetics in lower alpha-1-acid glycoprotein levels in humans. Front. Pharmacol. 12, 658039. 10.3389/fphar.2021.658039 33995081 PMC8116740

[B20] Park J. W. Park I. H. Kim J. M. Noh J. H. Kim K. A. Park J. Y. (2022). Rapid detection of FMO3 single nucleotide polymorphisms using a Pyrosequencing method. Mol. Med. Rep. 25, 48. 10.3892/mmr.2021.12564 34913068 PMC8674696

[B21] Schellong S. M. (2015). Dabigatran for the treatment of venous thromboembolism. Expert Rev. Hematol. 8, 413–425. 10.1586/17474086.2015.1052400 26111881

