# Supplementary material for: UGT2B15 single nucleotide polymorphism reduces dabigatran acylglucuronide formation in humans
Source: Front Pharmacol. 2025 Jan 9;15:1507915. doi: 10.3389/fphar.2024.1507915 (PMC11754044; doi:10.3389/fphar.2024.1507915)
Supplement: Supplementary file 1 [file Table1.docx]

**Supplementary table 1.** Comparisons of pharmacokinetic variables of free DAB by *ABCB1* and *CES1* genetic polymorphisms.

| **Parameters** | **Wild type (W)** | **Heterozygous (H)** | **Homozygous mutants (M)** | **H and M** | **P-value** | |
| --- | --- | --- | --- | --- | --- | --- |
| ***ABCB1* c.1236C>T (rs1128503)** | **CC (n = 24)** | **CT (n = 58)** | **TT (n = 42)** | **CT, TT (n = 100)** | **W vs H vs M** | **W vs H and M** |
| T_max_ (h) | 2.2 ± 0.5 | 2.4 ± 0.7 | 2.3 ± 0.8 | 2.3 ± 0.8 | 0.5061 | 0.4084 |
| C_max_ (ng/mL) | 107.7 ± 43.2 | 98 ± 46 | 105.2 ± 47.8 | 101 ± 46.7 | 0.6079 | 0.5219 |
| AUC_all_ (ng·h·mL^-1^) | 884.9 ± 372.1 | 863.6 ± 410 | 880.8 ± 393.3 | 870.8 ± 401.1 | 0.9657 | 0.8759 |
| Half-life (h) | 8.8 ± 0.9 | 9.2 ± 1.5 | 8.7 ± 1.4 | 9 ± 1.5 | 0.1471 | 0.5369 |
| CL/F (L/h) | 209 ± 124.5 | 221.8 ± 141.2 | 211.8 ± 120.8 | 217.6 ± 132.5 | 0.8945 | 0.7738 |
| ***ABCB1* c.2677G>T(A) (rs2032582)** | **GG (n = 18)** | **GA, TA, GT (n = 72)** | **AA, TT (n = 34)** | **GA, TA, GT, AA, TT (n = 106)** |  |  |
| T_max_ (h) | 2 ± 0.4 | 2.4 ± 0.8 | 2.3 ± 0.6 | 2.4 ± 0.7 | 0.0653 | **0.025*** |
| C_max_ (ng/mL) | 92.4 ± 52.3 | 104.4 ± 48.7 | 103.2 ± 35.9 | 104 ± 44.8 | 0.6103 | 0.3232 |
| AUC_all_ (ng·h·mL^-1^) | 768.3 ± 450 | 902.5 ± 414.8 | 868 ± 311.3 | 891.4 ± 383.5 | 0.4355 | 0.2219 |
| Half-life (h) | 8.8 ± 1.2 | 9 ± 1.5 | 8.9 ± 1.3 | 9 ± 1.4 | 0.7972 | 0.5778 |
| CL/F (L/h) | 250.9 ± 131.6 | 213.9 ± 137.9 | 201.8 ± 113.1 | 210 ± 130 | 0.933 | 0.2198 |
| ***ABCB1* c.3435C>T**  **(rs1045642)** | **CC (n = 52)** | **CT (n = 50)** | **TT (n = 22)** | **CT, TT (n = 72)** |  |  |
| T_max_ (h) | 2.2 ± 0.6 | 2.4 ± 0.8 | 2.3 ± 0.7 | 2.4 ± 0.8 | 0.3872 | 0.2037 |
| C_max_ (ng/mL) | 100.8 ± 52.9 | 101.1 ± 43.5 | 108.6 ± 33 | 103.4 ± 40.5 | 0.7830 | 0.7622 |
| AUC_all_ (ng·h·mL^-1^) | 861.5 ± 431.8 | 865.5 ± 393.3 | 920.5 ± 306.4 | 882.3 ± 367.7 | 0.8289 | 0.7735 |
| Half-life (h) | 9 ± 1.4 | 8.8 ± 1.5 | 9.1 ± 1.2 | 8.9 ± 1.4 | 0.6337 | 0.6144 |
| CL/F (L/h) | 225 ± 132.7 | 222.2 ± 146.5 | 180.3 ± 72.5 | 209.4 ± 129.4 | 0.3696 | 0.5137 |
| ***ABCB1 c.2482-2236G>A***  **(rs4148738)** | **GG (n = 25)** | **GA (n = 50)** | **AA (n = 49)** | **GA, AA (n = 99)** |  |  |
| T_max_ (h) | 2.3 ± 0.6 | 2.4 ± 0.8 | 2.3 ± 0.6 | 2.3 ± 0.7 | 0.5941 | 0.7719 |
| C_max_ (ng/mL) | 108.6 ± 35.3 | 101.6 ± 48.3 | 99.8 ± 48.6 | 100.7 ± 48.3 | 0.7306 | 0.4426 |
| AUC_all_ (ng·h·mL^-1^) | 914.5 ± 309.1 | 866.5 ± 410.5 | 859.9 ± 421.2 | 863.2 ± 413.7 | 0.8437 | 0.563 |
| Half-life (h) | 9.1 ± 1.3 | 8.8 ± 1.5 | 9 ± 1.3 | 8.9 ± 1.4 | 0.7079 | 0.5843 |
| CL/F (L/h) | 183.3 ± 77.5 | 224.9 ± 149.2 | 223.4 ± 131.3 | 224.2 ± 139.9 | 0.3791 |  |
| ***CES1* c.1168-33A>C**  **(rs2244613)** | **AA (n = 16)** | **AC (n = 65)** | **CC (n = 43)** | **AC, CC (n = 108)** |  |  |
| T_max_ (h) | 2.5 ± 0.8 | 2.2 ± 0.7 | 2.4 ± 0.7 | 2.3 ± 0.7 | 0.2205 | 0.3607 |
| C_max_ (ng/mL) | 109.9 ± 44.7 | 100 ± 49 | 102.9 ± 42.1 | 101.2 ± 46.2 | 0.7421 | 0.4818 |
| AUC_all_ (ng·h·mL^-1^) | 945.1 ± 394.4 | 848.2 ± 404.3 | 885.3 ± 384.1 | 863 ± 395 | 0.6628 | 0.4392 |
| Half-life (h) | 8.5 ± 0.8 | 9.2 ± 1.5 | 8.8 ± 1.2 | 9 ± 1.4 | 0.1475 | 0.12 |
| CL/F (L/h) | 201.1 ± 130.7 | 221.5 ± 125.7 | 213 ± 139.8 | 218.1 ± 130.9 | 0.8435 | 0.6285 |
| ***CES1* c.257 + 885T>C**  **(rs8192935)** | **TT (n = 78)** | **TC (n = 42)** | **GG (n = 4)** | **TC, GG (n = 46)** |  |  |
| T_max_ (h) | 2.4 ± 0.7 | 2.2 ± 0.7 | 2.5 ± 0.4 | 2.3 ± 0.7 | 0.6236 | 0.5009 |
| C_max_ (ng/mL) | 102.6 ± 44 | 99 ± 50.1 | 131 ± 36 | 101.8 ± 49.5 | 0.4147 | 0.9237 |
| AUC_all_ (ng·h·mL^-1^) | 885.2 ± 388.2 | 833.8 ± 414.9 | 1064.5 ± 278.6 | 853.9 ± 407.9 | 0.4923 | 0.6714 |
| Half-life (h) | 9.1 ± 1.4 | 8.8 ± 1.4 | 8.6 ± 0.7 | 8.7 ± 1.3 | 0.4419 | 0.21 |
| CL/F (L/h) | 209.5 ± 125.7 | 234.7 ± 142.9 | 145.5 ± 36.4 | 226.9 ± 139.1 | 0.3317 | 0.4735 |

C_max_, maximum concentration; T_max_, time to reach the maximum concentration; AUC_all_, total area under the plasma concentration–time curve; CL/F, oral clearance. *P < 0.05; ^a^P < 0.05 between W and H; ^b^P < 0.05 between W and M; ^c^P < 0.05 between H and M.

**Supplementary table 2.** Comparisons of pharmacokinetic variables of DABG by *ABCB1* and *CES1* genetic polymorphisms.

| **Parameters** | **Wild type (W)** | **Heterozygous (H)** | **Homozygous mutants (M)** | **H and M** | **P-value** | |
| --- | --- | --- | --- | --- | --- | --- |
| ***ABCB1* c.1236C>T (rs1128503)** | **CC (n = 24)** | **CT (n = 58)** | **TT (n = 42)** | **CT, TT (n = 100)** | **W vs H vs M** | **W vs H and M** |
| T_max_ (h) | 2.3 ± 0.6 | 2.1 ± 0.7 | 2.2 ± 0.7 | 2.2 ± 0.7 | 0.7540 | 0.4527 |
| C_max_ (ng/mL) | 41 ± 24.1 | 32.1 ± 17.7 | 33.7 ± 17.8 | 32.7 ± 17.6 | 0.1543 | 0.0586 |
| AUC_all_ (ng·h·mL^-1^) | 309 ± 204.5 | 247.7 ± 158.9 | 243.2 ± 147.9 | 245.8 ± 153.7 | 0.2442 | 0.0935 |
| Half-life (h) | 9.8 ± 2 | 10.5 ± 2.6 | 10.8 ± 2.5 | 10.6 ± 2.6 | 0.3372 | 0.1580 |
| ***ABCB1* c.2677G>T(A) (rs2032582)** | **GG (n = 18)** | **GA, TA, GT (n = 72)** | **AA, TT (n = 34)** | **GA, TA, GT, AA, TT (n = 106)** |  |  |
| T_max_ (h) | 2.1 ± 0.8 | 2.2 ± 0.7 | 2.2 ± 0.8 | 2.2 ± 0.7 | 0.6874 | 0.4459 |
| C_max_ (ng/mL) | 30.1 ± 15.6 | 36 ± 20.5 | 32.9 ± 18.2 | 35 ± 19.8 | 0.4504 | 0.3127 |
| AUC_all_ (ng·h·mL^-1^) | 220.1 ± 133.3 | 276.4 ± 181.2 | 239.2 ± 143.8 | 264.5 ± 173.7 | 0.3247 | 0.2949 |
| Half-life (h) | 10.5 ± 2.1 | 10.5 ± 2.8 | 10.4 ± 1.8 | 10.5 ± 2.5 | 0.9819 | 0.9037 |
| ***ABCB1* c.3435C>T**  **(rs1045642)** | **CC (n = 52)** | **CT (n = 50)** | **TT (n = 22)** | **CT, TT (n = 72)** |  |  |
| T_max_ (h) | 2.2 ± 0.7 | 2.1 ± 0.7 | 2.2 ± 0.7 | 2.1 ± 0.7 | 0.8747 | 0.6113 |
| C_max_ (ng/mL) | 33.4 ± 20.9 | 34.9 ± 18.4 | 35.4 ± 17.6 | 35 ± 18.1 | 0.8953 | 0.6452 |
| AUC_all_ (ng·h·mL^-1^) | 250.1 ± 177.3 | 268.6 ± 165.7 | 252.9 ± 141.2 | 263.8 ± 157.8 | 0.8444 | 0.6518 |
| Half-life (h) | 10.5 ± 3.3 | 10.5 ± 1.7 | 10.2 ± 1.5 | 10.4 ± 1.6 | 0.8934 | 0.7998 |
| ***ABCB1 c.2482-2236G>A***  **(rs4148738)** | **GG (n = 25)** | **GA (n = 50)** | **AA (n = 49)** | **GA, AA (n = 99)** |  |  |
| T_max_ (h) | 2.2 ± 0.8 | 2.1 ± 0.7 | 2.2 ± 0.7 | 2.2 ± 0.7 | 0.7110 | 0.8359 |
| C_max_ (ng/mL) | 33.4 ± 17.7 | 34.1 ± 18.3 | 35.1 ± 21.1 | 34.6 ± 19.7 | 0.9341 | 0.7835 |
| AUC_all_ (ng·h·mL^-1^) | 237 ± 140.2 | 263.4 ± 162.8 | 263.3 ± 182.1 | 263.3 ± 171.8 | 0.7804 | 0.4804 |
| Half-life (h) | 10.4 ± 1.5 | 10.6 ± 2.3 | 10.4 ± 3 | 10.5 ± 2.7 | 0.9517 | 0.9571 |
| ***CES1* c.1168-33A>C**  **(rs2244613)** | **AA (n = 16)** | **AC (n = 65)** | **CC (n = 43)** | **AC, CC (n = 108)** |  |  |
| T_max_ (h) | 2.1 ± 0.6 | 2.1 ± 0.7 | 2.2 ± 0.7 | 2.2 ± 0.7 | 0.7224 | 0.7695 |
| C_max_ (ng/mL) | 35.1 ± 20.5 | 34.2 ± 20.3 | 34.3 ± 17.5 | 34.2 ± 19.1 | 0.9859 | 0.8665 |
| AUC_all_ (ng·h·mL^-1^) | 289.2 ± 197.6 | 253.2 ± 175.3 | 253.8 ± 138.7 | 253.4 ± 161 | 0.7256 | 0.4224 |
| Half-life (h) | 10.2 ± 2.1 | 10.8 ± 3 | 10.1 ± 1.6 | 10.5 ± 2.5 | 0.2938 | 0.6195 |
| ***CES1* c.257 + 885T>C**  **(rs8192935)** | **TT (n = 78)** | **TC (n = 42)** | **GG (n = 4)** | **TC, GG (n = 46)** |  |  |
| T_max_ (h) | 2.2 ± 0.7 | 2.2 ± 0.7 | 2.1 ± 0.6 | 2.2 ± 0.7 | 0.9897 | 0.9975 |
| C_max_ (ng/mL) | 35.2 ± 18.9 | 31.5 ± 18.9 | 46.8 ± 27.4 | 32.9 ± 19.8 | 0.2586 | 0.5185 |
| AUC_all_ (ng·h·mL^-1^) | 260.3 ± 160.6 | 245.2 ± 174.5 | 349.5 ± 183.4 | 254.3 ± 175.7 | 0.4796 | 0.8467 |
| Half-life (h) | 10.2 ± 2.1 | 11 ± 3.2 | 9.7 ± 0.7 | 10.9 ± 3 | 0.2573 | 0.1763 |

C_max_, maximum concentration; T_max_, time required to reach the maximum concentration; AUC_all_, total area under the plasma concentration–time curve. *P < 0.05; ^a^P < 0.05 between W and H; ^b^P < 0.05 between W and M; ^c^P < 0.05 between H and M.

**Supplementary table 3.** Comparisons of metabolite-parent ratios (m/p ratio) for DAB by *ABCB1* and *CES1* genetic polymorphisms.

| **Parameters** | **Wild type (W)** | **Heterozygous (H)** | **Homozygous mutants (M)** | **H and M** | **P-value** | |
| --- | --- | --- | --- | --- | --- | --- |
| ***ABCB1* c.1236C>T (rs1128503)** | **CC (n = 24)** | **CT (n = 58)** | **TT (n = 42)** | **CT, TT (n = 100)** | **P-value** | |
| C_max_ m/p ratio | 0.4 ± 0.2 | 0.3 ± 0.1 | 0.3 ± 0.1 | 0.3 ± 0.1 | 0.4601 | 0.2249 |
| AUC_all_ m/p ratio | 0.4 ± 0.2 | 0.3 ± 0.1 | 0.3 ± 0.1 | 0.3 ± 0.1 | 0.2264 | 0.0842 |
| ***ABCB1* c.2677G>T(A) (rs2032582)** | **GG (n = 18)** | **GA, TA, GT (n = 72)** | **AA, TT (n = 34)** | **GA, TA, GT, AA, TT (n = 106)** |  |  |
| C_max_ m/p ratio | 0.4 ± 0.2 | 0.4 ± 0.1 | 0.3 ± 0.1 | 0.3 ± 0.1 | 0.3264 | 0.362 |
| AUC_all_ m/p ratio | 0.3 ± 0.2 | 0.3 ± 0.1 | 0.3 ± 0.1 | 0.3 ± 0.1 | 0.3556 | 0.3429 |
| ***ABCB1* c.3435C>T**  **(rs1045642)** | **CC (n = 52)** | **CT (n = 50)** | **TT (n = 22)** | **CT, TT (n = 72)** |  |  |
| C_max_ m/p ratio | 0.4 ± 0.2 | 0.4 ± 0.1 | 0.3 ± 0.1 | 0.3 ± 0.1 | 0.7840 | 0.8308 |
| AUC_all_ m/p ratio | 0.3 ± 0.2 | 0.3 ± 0.1 | 0.3 ± 0.1 | 0.3 ± 0.1 | 0.4746 | 0.9485 |
| ***ABCB1* c.2482-2236G>A**  **(rs4148738)** | **GG (n = 25)** | **GA (n = 50)** | **AA (n = 49)** | **GA, AA (n = 99)** |  |  |
| C_max_ m/p ratio | 0.3 ± 0.1 | 0.4 ± 0.1 | 0.4 ± 0.2 | 0.4 ± 0.2 | 0.2432 | 0.1038 |
| AUC_all_ m/p ratio | 0.3 ± 0.1 | 0.3 ± 0.1 | 0.3 ± 0.2 | 0.3 ± 0.2 | 0.1775 | 0.063 |
| ***CES1* c.1168-33A>C**  **(rs2244613)** | **AA (n = 16)** | **AC (n = 65)** | **CC (n = 43)** | **AC, CC (n = 108)** |  |  |
| C_max_ m/p ratio | 0.3 ± 0.1 | 0.4 ± 0.1 | 0.4 ± 0.2 | 0.4 ± 0.1 | 0.8181 | 0.5276 |
| AUC_all_ m/p ratio | 0.3 ± 0.2 | 0.3 ± 0.1 | 0.3 ± 0.1 | 0.3 ± 0.1 | 0.9629 | 0.7955 |
| ***CES1* c.257 + 885T>C**  **(rs8192935)** | **TT (n = 78)** | **TC (n = 42)** | **GG (n = 4)** | **TC, GG (n = 46)** |  |  |
| C_max_ m/p ratio | 0.4 ± 0.2 | 0.3 ± 0.1 | 0.3 ± 0.1 | 0.3 ± 0.1 | 0.7086 | 0.4156 |
| AUC_all_ m/p ratio | 0.3 ± 0.1 | 0.3 ± 0.2 | 0.3 ± 0.1 | 0.3 ± 0.2 | 0.9774 | 0.9102 |

C_max_, maximum concentration; AUC_all_, total area under the plasma concentration–time curve. *P < 0.05; ^a^P < 0.05 between W and H; ^b^P < 0.05 between W and M; ^c^P < 0.05 between H and M.
